# Barratt Impulsivity in Healthy Adults Is Associated with Higher Gray Matter Concentration in the Parietal Occipital Cortex that Represents Peripheral Visual Field

**DOI:** 10.3389/fnhum.2017.00222

**Published:** 2017-05-04

**Authors:** Jaime S. Ide, Hsiang C. Tung, Cheng-Ta Yang, Yuan-Chi Tseng, Chiang-Shan R. Li

**Affiliations:** ^1^Department of Psychiatry, Yale University School of MedicineNew Haven, CT, USA; ^2^Department of Psychology, National Cheng Kung UniversityTainan, Taiwan; ^3^Department of Industrial Design, National Cheng Kung UniversityTainan, Taiwan; ^4^Department of Neuroscience, Yale UniversityNew Haven, CT, USA; ^5^Interdepartmental Neuroscience Program, Yale UniversityNew Haven, CT, USA; ^6^Beijing Huilongguan HospitalBeijing, China

**Keywords:** impulse control, inattention, visual cortex, parieto-occipital cortex, area V6, area PO

## Abstract

Impulsivity is a personality trait of clinical importance. Extant research focuses on fronto-striatal mechanisms of impulsivity and how executive functions are compromised in impulsive individuals. Imaging studies employing voxel based morphometry highlighted impulsivity-related changes in gray matter concentrations in a wide array of cerebral structures. In particular, whereas prefrontal cortical areas appear to show structural alterations in individuals with a neuropsychiatric condition, the findings are less than consistent in the healthy population. Here, in a sample (*n* = 113) of young adults assessed for Barratt impulsivity, we controlled for age, gender and alcohol use, and showed that higher impulsivity score is associated with increased gray matter volume (GMV) in bilateral medial parietal and occipital cortices known to represent the peripheral visual field. When impulsivity components were assessed, we observed that this increase in parieto-occipital cortical volume is correlated with inattention and non-planning but not motor subscore. In a separate behavioral experiment of 10 young adults, we demonstrated that impulsive individuals are more vulnerable to the influence of a distractor on target detection in an attention task. If replicated, these findings together suggest aberrant visual attention as a neural correlate of an impulsive personality trait in neurotypical individuals and need to be reconciled with the literature that focuses on frontal dysfunctions.

## Introduction

Impulsivity has consistently been linked to neuropsychiatric conditions such as addiction and attention deficit hyperactivity disorder (ADHD, Bari and Robbins, 2013). Extant research focuses largely on executive dysfunction to elucidate the neurobiological basis of impulsivity. In particular, a host of brain imaging studies characterized how regional brain activations and functional connectivities are altered in behavioral tasks of executive functions to address impulsivity in individuals with or without a neurological diagnosis (Dalley et al., 2008; Cocchi et al., 2012; Farr et al., 2012; Friederich et al., 2013; Fineberg et al., 2014). This preponderant body of work has advanced our understanding of the fronto-striatal mechanisms of cognitive control and influenced research of cognitive enhancement to mitigate impulsive behavior.

Structural imaging provides another tool to investigate the cerebral correlates of impulsivity. For instance, voxel-based morphometry (VBM) showed decreased caudate gray matter volume (GMV) in association with hyperactive and impulsive symptoms in male adults with ADHD (Onnink et al., 2014). Compared to healthy controls, individuals with borderline personality disorders showed reductions in GMV in ventral cingulate gyrus and medial temporal lobe, a difference that appears to be accounted for by impulsivity (Soloff et al., 2008). GMV of the anterior cingulate cortex is negatively correlated with impulsivity as assessed with the Barratt Impulsivity Scale (BIS-11) in individuals at risk for psychosis (Lee et al., 2013) and in patients with bipolar disorder (Matsuo et al., 2009b). Compared with healthy subjects, individuals addicted to online gambling showed significant gray matter atrophy in the right orbitofrontal cortex, bilateral insula and right supplementary motor area (Weng et al., 2013). In treatment-seeking codeine-containing cough syrup users, Barratt impulsivity is associated with decreased GMV of bilateral ventromedial prefrontal cortex (Qiu et al., 2014). In non-treatment-seeking cocaine users, Barratt impulsivity is associated with reduced GMV in the right orbitofrontal cortex, left precentral gyrus and right superior frontal gyrus (Crunelle et al., 2014). Other studies have associated altered cerebral GMV’s with impulsivity as evaluated by performance in the go/nogo (Schiffer et al., 2010; O’Callaghan et al., 2013; Depue et al., 2014), stop signal (Tabibnia et al., 2011), Stroop (Moreno-López et al., 2012b; Albein-Urios et al., 2013), target detection/continuous performance (Liu et al., 2013), verbal inhibition (O’Callaghan et al., 2013) and Wisconsin Card Sorting (Schiffer et al., 2010) tasks in various clinical populations.

In healthy individuals, Cloninger’s impulsiveness score is inversely associated with GM volume in left orbitofrontal cortex in adolescents (Schilling et al., 2013). Emotion-based rash impulsivity is associated with smaller GMV in the dorsomedial prefrontal cortex and right temporal pole (Muhlert and Lawrence, 2015). Barratt impulsivity has been linked to decreased GMV of bilateral orbitofrontal cortex (Matsuo et al., 2009a) and to decreased cortical thickness of the left middle frontal gyrus, orbitofrontal and superior frontal gyri as well as increased cortical thickness of the right inferior temporal cortex (Schilling et al., 2012). In healthy young adults assessed with a delayed discounting task, both non-planning impulsivity and greater steepness of discounting are associated with increased GMV in the medial and middle frontal gyrus, anterior/middle cingulate cortices and the orbitofrontal cortex (Cho et al., 2013). In addition, steeper discounting is associated with decreased GMV in bilateral ventral putamen.

Together, whereas impulsivity is predominantly associated with decreased prefrontal cortical GMV in neuropsychiatric conditions, the findings appear to be less consistent in healthy individuals. One needs to consider differences in subject characteristics and the use of various instruments (e.g., questionnaire vs. behavioral tests) in examining the discrepancy amongst the studies. Furthermore, few studies have controlled for age and alcohol use, both of which are known to affect cerebral morphometry (Hu et al., 2014; Ide et al., 2014, 2017).

In the current study, we aimed to identify the cerebral structural correlates of Barratt Impulsivity in a large sample of healthy individuals. Specifically, we controlled for age, gender and drinking variables in a multiple regression and hypothesized that impulsivity will be associated with reduced GM volume in the prefrontal cortex.

## Materials and Methods

### Subjects and Assessment

One hundred and thirteen healthy young adults (66 women; age 32 ± 14 years; all right-handed) were paid to participate in the study. Participants were recruited from the greater New Haven area. All participants were screened to be free of major medical illness, past or present neurological and psychiatric illnesses including substance use disorders using Structured Clinical Interview for DSM Disorders (First et al., 1995). All participants denied current use of illicit substance and showed negative urine toxicology tests for stimulants, opioids, marijuana and benzodiazepines at the time of initial screening and MRI. Individuals who were using any psychotropic medications were not invited to participate in the study. Pregnant or lactating women were also excluded. Participants were further required to be free of MRI-contraindications based on the Yale Magnetic Resonance Research Center’s safety guidelines. All participants gave written informed consent following a protocol approved by the Yale Human Investigation Committee.

All participants completed assessment of impulsivity on the BIS-11 (Patton et al., 1995). The BIS-11 is a 30-item self-report questionnaire designed to measure impulsivity. All items are scored on a 4-point scale (1 = rarely/never; 2 = occasionally; 3 = often; 4 = almost always/always). The total score thus ranges from 30 to 120, with a higher score indicating increased impulsivity. Eleven of the 30 items are reverse scored to avoid response bias. The BIS-11 has been translated into Chinese, Italian and Japanese with good internal consistency and test–retest reliability (Fossati et al., 2001; Someya et al., 2001; Li and Chen, 2007). Factor analysis revealed three independent components in the BIS-11 (Patton et al., 1995): attentional impulsiveness (assessing the ability to focus on the task at hand); motor impulsiveness (assessing the tendency to act on the spur of the moment); and non-planning impulsiveness (assessing the tendency to plan and think carefully). The total score of BIS-11 of the participants averaged 59.8 ± 9.2 (mean ± SD), with 14.5 ± 3.4, 22.1 ± 3.4 and 23.2 ± 4.27 each for inattention, motor and non-planning subscore. Because alcohol use has been consistently linked to changes in cerebral morphometry, all participants were also assessed with questionnaires regarding their alcohol use over the past year, including the average number of days of alcohol use and average number of drinks consumed per occasion, framed on a monthly basis. Across all participants, monthly frequency of drinking and the number of drinks consumed in a single occasion each averaged at 4.5 ± 5.0 and 2.2 ± 1.6 (mean ± SD). These variables of alcohol use, age and gender were included as covariates in data analysis.

### Imaging Protocol

Participants were scanned on a Siemens 3-Tesla scanner (Trio; Siemens AG, Erlangen, Germany). Data for each participant consisted of a single high-resolution T1-weighted gradient-echo scan: 176 slices; 1 mm^3^ isotropic voxels; field of view = 256 × 256 mm; data acquisition matrix = 256 × 256; Repetition Time = 2530 ms; Echo Time = 3.66 ms, bandwidth = 181 Hz/pixel; flip angle = 7°.

### Voxel-Based Morphometry (VBM)

The aim of VBM is to identify differences in the local composition of brain tissue and its association with behavioral and cognitive measures, while discounting large scale differences in gross anatomy and position. This can be achieved by spatially normalizing individuals’ structural images to the same stereotactic space, segmenting the normalized images into distinct brain tissues, smoothing the gray-matter images, and performing a statistical test to localize significant associations between anatomy and measures of interest (Ashburner and Friston, 2000).

VBM was performed using the VBM8 toolbox^1^ packaged in Statistical Parametric Mapping 8 (Wellcome Department of Imaging Neuroscience, University College London, U.K.). T_1_-images were first co-registered to the Montreal Neurological Institute or MNI template space (1.5 mm^3^ isotropic voxels) using a multiple stage affine transformation, during which the 12 parameters were estimated. Co-registration started with a coarse affine registration using mean square differences, followed by a fine affine registration using mutual information. Coefficients of the basis-functions that minimize the residual square difference (between individual image and the template) were estimated. Tissue probability maps constructed from 471 healthy subjects were used in affine transformation. After affine transformation, T_1_-images were corrected for intensity bias field (kernel size FWHM = 60 mm) and a local means denoising filter (Manjón et al., 2010) with default parameter 1 was applied to account for intensity variations (inhomogeneity) and noise caused by different positions of cranial structures within the MRI coil. Finally, they were segmented into cerebrospinal fluid, gray and white-matters, using an adaptive maximum a posteriori method (Rajapakse et al., 1997) with *k*-means initializations, as implemented in VBM8, generating tissue class maps (which included the gray matter or GM maps). In segmentation, partial volume estimation was performed with default parameter 5, with a simplified mixed model of at most two tissue types (Tohka et al., 2004). Segmented and the initially registered tissue class maps were normalized using DARTEL (Ashburner, 2007), a fast diffeomorphic image registration algorithm of SPM. As a high-dimensional non-linear spatial normalization method, DARTEL generates mathematically consistent inverse spatial transformations. We used the standard DARTEL template in MNI space, constructed from 550 healthy subjects of the IXI-database^2^, to drive DARTEL normalization. Normalized GM maps were modulated to obtain the absolute volume of GM tissue corrected for individual brain sizes. The GM maps were smoothed by convolving with an isotropic Gaussian kernel. Smoothing helps to compensate for the inexact nature of spatial normalization and reduces the number of statistical comparisons (thus making the correction for multiple comparisons less severe); however, it reduces the accuracy of localization. Most VBM studies used a kernel size of FWHM = 12 mm. We used a smaller kernel size of FWHM = 8 mm to enhance localization accuracy.

In group analyses, we regressed the GM volumes of the whole brain against the BIS-11 score, with drinking variables (average monthly frequency of drinking and the average number of drinks consumed in a single occasion), age and gender as covariates. The results were examined with voxel *p* < 0.001 uncorrected combined with a cluster threshold *p* < 0.05, FWE corrected. In order to highlight the location of the clusters identified from group analyses, we overlaid the clusters on an inflated brain. First, we resampled the cluster volumes to Freesurfer’s average MNI template space to account for orientation and resolution differences. We then projected the volume to surface using Freesurfer’s function mri_vol2surf, which identifies the nearest neighbor point between the white and pial surfaces (Fischl et al., 1999). We used a fractional projection along surface normal (depth) of 0.5, thus defining the surface in the center of gray matter.

### A Behavioral Experiment

We conducted an independent experiment to examine the potential influence of impulsivity on attention. Ten students (5 men, 20.1 ± 1.32 years of age, right-handed and with normal or corrected-to-normal vision) from the National Cheng Kung University completed the BIS-11 questionnaire (Li and Chen, 2007) and a behavioral experiment. All participants were compensated for their time and signed a written informed consent prior to the study according to institute guidelines.

The experiment was conducted in a dimly lit room. A personal computer with a 3.20 G-Hz Intel Core I5 processor controlled stimulus display and recorded the participants’ responses. The visual stimuli were presented on a 19-inch CRT monitor (CTX VL951T) with a refresh rate of 85 Hz and 1024 (width) × 768 (height) in pixel resolution, at a viewing distance of 60 cm. A chin-rest was used to prevent head movements. An Eyelink 1000 eye tracker (SR Research, Mississauga, ON, Canada) with a sampling rate of 1000 Hz was used to monitor eye movements.

In the attention task, participants were to decide whether a target event occurred. A trial started with presentation of an asterisk (0.5° in visual angle and of gray color (*x* = 0.295, *y* = 0.315, luminance = 2.87 cd/m^2^, RGB: 190, 190, 190) for 300 ms at the center of visual display. In half of the trials (target-present trials), the asterisk dimmed (*x* = 0.294, *y* = 0.314, luminance = 2.36 cd/m^2^, RGB: 180, 180, 180) for 200 ms randomly between 364 and 600 ms after its onset, and resumed its luminance and stayed on for another 300 ms. The asterisk did not dim in the other half of trials—target-absent trials. In 1/3 of all trials, a “distractor” (a disk, 1.5° in visual angle and of white color (*x* = 0.293, *y* = 0.313, luminance = 5.93 cd/m^2^, RGB: 250, 250, 250) appeared for 64 ms before asterisk dimming at one of four peripheral locations 6° from the center (upper left, upper right, lower left and lower right, randomly). A distractor also occurred in target-absent trials with timings matching those of the target-present trials. Participants were instructed to ignore the distractor and responded to the prompt (dimming YES or NO) and rated their confidence level on a scale from 1 (not confident at all) to 4 (very confident).

After a practice run, participants performed a total of 1200 trials, divided into 20 blocks, with a short break in between blocks. Thus, there were a total of 400 target+/distractor−, 200 target+/distractor+, 400 target−/distractor− and 200 target−/distractor+ trials.

## Results

### Barratt Impulsivity and Gray Matter Volumes

**Figure 1:**
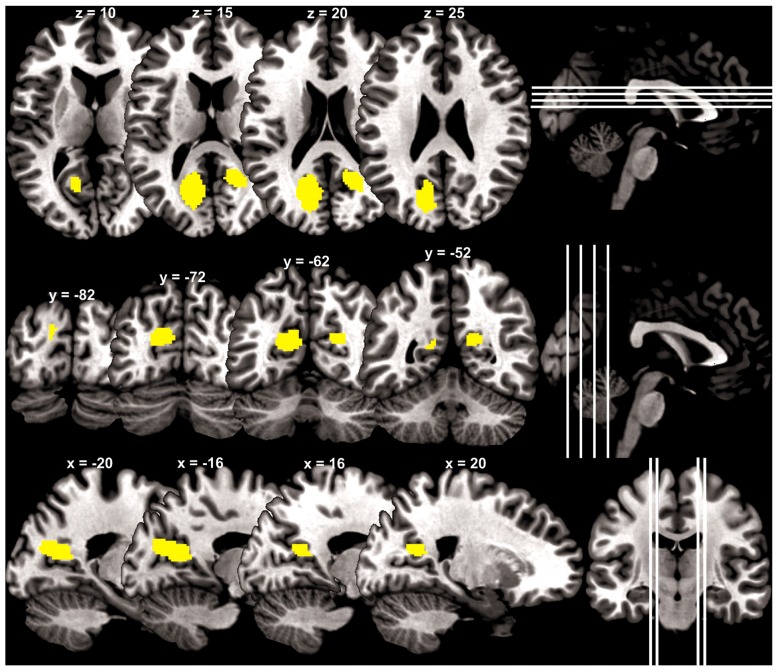
**Brain regions (yellow) with gray matter volume (GMV) increasing with impulsivity overlaid on a structural template in axial, coronal and parasagittal sections**.

At a cluster threshold of *p* < 0.05, corrected for family-wise error of multiple comparisons, GMV were positively correlated with Barratt impulsivity in the left parieto-occipital sulcus (5454 mm^3^, peak MNI coordinate [−20 −57 15], *Z* = 4.90) and right parieto-occipital sulcus (1819 mm^3^, peak MNI coordinate [21 −51 18], *Z* = 4.37; Figure 1).

**Figure 2:**
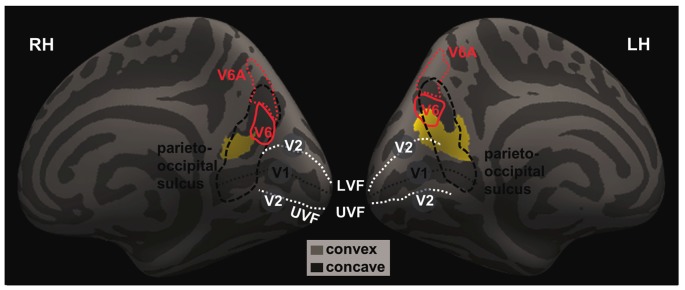
**The projected left and right GM volumes are overlaid on inflated left (LH) and right (RH) hemispheres of the Freesurfer’s fsaverage surface**. The parieto-occipital sulcus (thick dashed black line) runs downward on the medial surface and joins the calcarine fissure (thin dashed black line) below, marking the boundary between the precuneus and cuneus and between the parietal and occipital lobes. In humans, area V6 occupies the most dorsal part of the parieto-occipital sulcus (Pitzalis et al., 2015). The medial and lateral parts of V6 each represents the peripheral and central visual fields; and the dorsal and ventral parts of the V6 each represents the upper and lower visual fields. The white dashed lines mark the approximate boundaries between V1 and V2. The lower/upper visual fields of V1 and V2 are represented in the dorsal/ventral bank of the calcarine sulcus. The central visual fields of V1 and V2 and represented toward the occipital pole while the peripheral visual fields are represented anteriorly in adjacence to the parieto-occipital sulcus. Thus, it appears that the identified clusters are both located in the occipital and parietal cortices that represent the lower, peripheral visual fields.

We highlighted the location of these two clusters with respect to brain regions in and around the parieto-occipital sulci by overlaying the clusters on an inflated brain following a previous work that mapped the human visual area V6 and V6A (Figure 2; Pitzalis et al., 2013), as described in “Materials and Methods” Section.

**Figure 3:**
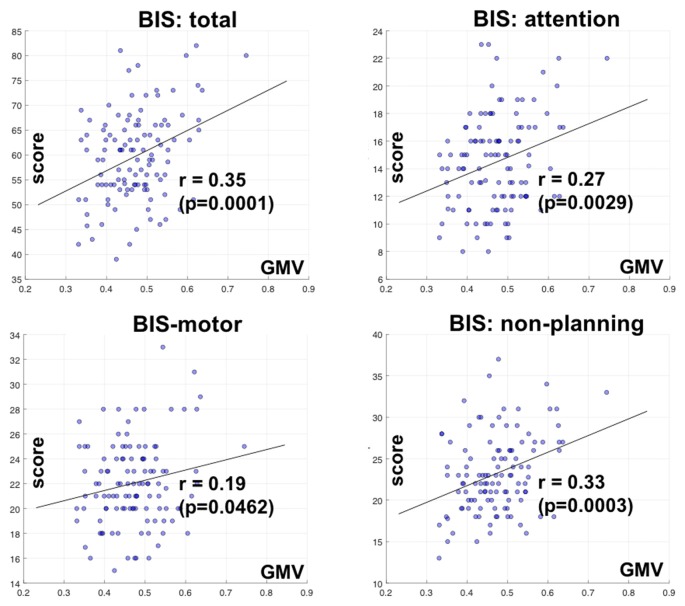
**Correlation of GMV of the cluster identified in bilateral parietal occipital cortex with Barratt impulsivity total and subscores**. *R* and *P* values are shown for Pearson regression. Each circle represents the data of one participant.

We also correlated the GMV of the identified clusters with the BIS-11 subscores with linear regression. The GMV of parieto-occipital clusters was significantly correlated with inattention and non-planning subscores (*p* < 0.0029 and *p* < 0.0003, respectively), but not motor subscore (*p* < 0.0462) after correcting for multiple comparisons (Figure 3).

### Barratt Impulsivity and Distractor Effects on Attention

**Figure 4:**
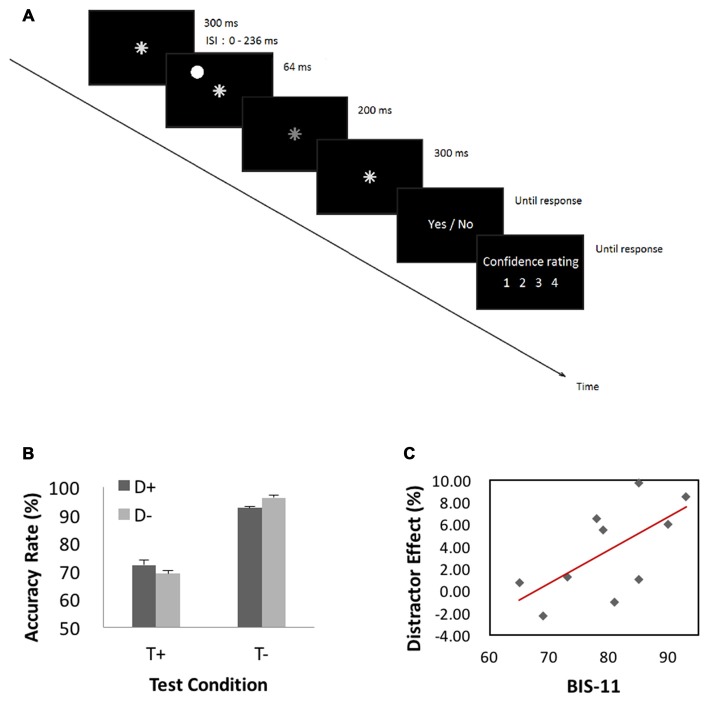
**(A)** Behavioral paradigm of the attention task. A trial started with presentation of an asterisk for 300 ms at the center of visual display. In half of the trials (target-present trials), the asterisk dimmed for 200 ms randomly between 364 and 600 ms after its onset, and resumed its luminance and stayed on for another 300 ms. The asterisk did not dim in the other half of trials—target-absent trials. In 1/3 of all trials, a “distractor” appeared for 64 ms before asterisk dimming at one of four peripheral locations 6° from the center (upper left, upper right, lower left and lower right, randomly). A distractor also occurred in target-absent trials with timings matching those of the target-present trials. Participants were instructed to ignore the distractor and responded to the prompt (dimming YES or NO) and rated their confidence level on a scale from 1 (not confident at all) to 4 (very confident). **(B)** Performance results. A repeated measures ANOVA on accuracy rate showed a significant main effect of target condition with higher accuracy for target-absent than target-present trials. **(C)** Correlation with BIS-11 score. We quantified the influence of distractor by subtracting the accuracy rate of distractor-present from that of distractor-absent, each for target-present and target-absent trials. This distractor effect was significantly correlated with BIS-11 score for target-absent but not target-present trials.

The behavioral paradigm is illustrated in Figure 4A. Figure 4B shows performance results. We conducted a 2 (target presence/absence) × 2 (distractor presence/absence) repeated measures ANOVA on accuracy rate. The results showed a significant main effect of target condition (*F*_(1,9)_ = 11.23, MSE = 1.22, *p* < 0.05, $\eta_{\text{p}}^{\text{2}}$ = 0.92), with higher accuracy for target-absent than target-present trials. Neither distractor main effect (*p* > 0.70) nor interaction effect (*p* > 0.08) was significant. Because target-present and target-absent trials differed significantly in accuracy rate and previous studies suggested that “sameness” judgments involve different processes from “difference” judgments in a same-different judgment task (Egeth, 1966; Farell, 1985), we quantified the influence of distractor by subtracting the accuracy rate of distractor-present from that of distractor-absent, each for target-present (−3.0 ± 7.4%) and target-absent (4.0 ± 4.2%) trials. In a linear regression, we showed that this distractor effect was correlated with BIS-11 score for target-absent (*r* = 0.65, *p* < 0.05, Pearson regression, Figure 4C) but not for target-present (*r* = −0.4, *p* > 0.26) trials.

In an additional analysis, we employed receiver operating characteristic (ROC) analysis by fitting an equal-variance signal detection model (Green and Swets, 1966) to the confidence ratings of each individual subject. Using a maximum likelihood method, detection sensitivity (d′), a bias-free measure, was estimated each for distractor-present and distractor-absent condition. The d′ was 2.45 ± 1.23 (mean ± SD) and 2.48 ± 1.20 for the distractor-present and distractor-absent condition (*p* > 0.9, paired-sample *t* test), respectively. Likewise, we quantified the distractor effect by subtracting d′ of the distractor-present condition from that of the distractor-absent condition. In a linear regression, BIS-11 score showed marginally significant correlation with d′ distractor effect (*r* = 0.60, *p* < 0.06).

## Discussion

Contrary to our hypothesis, we found that Barratt impulsivity is associated with higher GMV in areas of the parieto-occipital sulcus bilaterally. An overlay of these clusters on an inflated brain image indicates that they are mostly located in parietal-occipital sulcus of medial parietal and occipital cortex that represents the lower peripheral visual field, covering part of the area V6 (Stenbacka and Vanni, 2007; Henriksson et al., 2012; Pitzalis et al., 2013, 2015). Further, in a separate experiment, we observed that individuals with higher Barratt impulsivity are more vulnerable to the influence of a distractor on target detection in an attention task. Together, these findings suggest the need of more studies to investigate attention deficit and increased parieto-occipital gray matter representation of the peripheral visual field as a mechanism of Barratt impulsivity.

It is not exactly clear why Barratt impulsivity was not associated with changes in frontal cortical GMV in the current study. However, as we noted earlier, studies varied with respect with the extent of frontal cortical involvement and the exact locations where changes in GMV were observed. It is possible that a combination of various factors contributes to this discrepancy in findings, including the various instruments and behavioral tasks used to evaluate impulsivity. For instance, self-report and behavioral measures of impulsivity may not be correlated (Reynolds et al., 2006). A recent study examined the latent structures of impulsivity assessed with a repertoire of widely employed instruments, including the Delay Discounting Task, Monetary Choice Questionnaire, Conners’ Continuous Performance Test, Go/NoGo Task, Stop Signal Task, Barratt Impulsiveness Scale and the UPPS-P Impulsive Behavior Scale (MacKillop et al., 2016). The findings suggested that diverse measures of impulsivity can broadly be organized into three categories—impulsive choice, impulsive action and impulsive personality traits—that are largely distinct from one another. That is, individual instruments may capture distinct aspects of impulsivity and their underlying neural circuits. These considerations suggest the importance of qualifying the current morphometric and behavioral findings as specific to Barratt impulsivity.

### Impulsivity and Visual Cortical Representations

Most imaging work to map the visual field representation focused on foveal and perifoveal vision. A few studies specifically examined the cortical representation of the peripheral visual field, with stimuli presented far out to 50° of visual angle. These studies showed that the central visual field is represented in the posterior part of occipital cortex, close to the occipital pole (Stenbacka and Vanni, 2007; Hoffmann et al., 2009). In contrast, the peripheral visual field is represented in the anterior part of the occipital cortex, in close proximity to the parieto-occipital sulcus and area V6 (Pitzalis et al., 2015). According to one study, in stereotaxic coordinates, visual angles within 12° are represented at *x* = 12, *y* = −78 to −84, *z* = 7 –10 on the right hemisphere, and *x* = −6, *y* = −79 to −84, *z* = −6 to 4 on the left hemisphere. Visual angles of 30–49° are represented at *x* = 15, *y* = −52 to −66, *z* = 3–20 on the right hemisphere, and *x* = −21 to −18, *y* = −58 to −69, *z* = 6–15 on the left hemisphere (Stenbacka and Vanni, 2007). In both cases, the representation of the lower visual field sits dorsal to that of the upper field (Wu et al., 2012).

The cluster in the left hemisphere overlaps with the ventral part of Area V6, which along with Area V6A and other visual cortical areas has recently been mapped topographically (Pitzalis et al., 2013, 2015). Area V6 receives ascending inputs from the peripheral field representation of visual areas V2 and V3, and is heavily connected to areas MST and 7a, as well as other regions in the intraparietal sulcus, premotor cortex and superior colliculus, suggesting its role in sensorimotor integration to support allocentric coding of heading direction and outward reaching movements (Shipp et al., 1998; Sulpizio et al., 2015). The peripheral visual field representation is larger in humans than in monkeys (Adams et al., 2007; Wu et al., 2012), perhaps to facilitate visual motor interaction including eye hand coordination. In ventral and lateral occipital cortex, attentional enhancement of visual responses was greater for central compared to peripheral eccentricities, whereas the opposite pattern was observed in dorsal stream areas, where attentional enhancement of positive fMRI responses was greater in the visual periphery (Bressler et al., 2013). There also appears to be hemispheric differences in visual and attentional functions with individual differences in the extent of lateralization across hemispheres (Thiebaut de Schotten et al., 2011; Szczepanski and Kastner, 2013). For instance, studies of human left but not right hemispheric parietal visual field representations showed that attention to the stimulus is associated with preferred locations of the population receptive field to move further to the right (i.e., toward the contralateral periphery; Sheremata and Silver, 2015). The latter finding suggests that attention to the right hemispheric personal space serves to further facilitate response to the farther visual periphery. One is tempted to speculate that, if increased GMV in the peripheral representation of the parieto-occipital sulcus is a structural correlate of this functional property, impulsive individuals may be particularly vulnerable to distraction by stimuli during exploration in the right peripersonal space.

We did not examine attentional functions in the cohort of subjects who participated in imaging; thus, the interpretation of the VBM findings remains tentative and needs to be confirmed in future work. However, in an independent study, we showed that impulsivity is positively correlated with the magnitude of distractor effect on target detection in an attention task. A significant correlation was observed only for target-absent trials likely because target-absent decisions involve more confirmation processes and thus are more vulnerable to distraction by a peripheral stimulus. Further, additional analyses based on signal detection theory showed that the distractor affected target detection sensitivity rather than response bias. Together, these findings suggested that impulsive individuals are more prone to distractor effect in this attention task.

Landau et al. (2012) showed that participants with high Barratt impulsivity exhibited larger involuntary attention effects in a spatial cueing task. In this study of a reaction time (RT) task, a cue predicted or did not predict the target location in alternating blocks and RT was measured to cued and uncued targets. Compared to low impulsivity individuals, high impulsivity individuals are influenced less by predictive but more by nonpredictive spatial cueing in RT. Further, in a flankers task in which the flanker stimuli are associated or not associated with target identity, high impulsivity individuals are particularly vulnerable to the distracting effects of the irrelevant flanker stimuli. The authors suggested that Barratt impulsivity is associated with a wider spread of spatial attention to account for the findings from the two experiments.

### Potential Clinical Relevance

The clinical relevance of the current findings remains to be established. Whereas earlier studies focused on the relationship between executive dysfunction and altered prefrontal structure and function, the current results suggest the importance of exploring attentional processes as a mechanism underlying impulsivity and various psychopathologies that implicate impulsivity. This issue may be particularly relevant to ADHD where clinical diagnosis distinguishes inattention and hyperactivity subtypes (Li et al., 2004). Boys with ADHD were shown to demonstrate increased GMV in the right occipital cortex, although the locus of structural alteration (*x* = 20, *y* = −86, *z* = 29) did not mirror the current findings (Wang et al., 2007). Further, earlier studies reported reduced GMV in early visual cortical areas (Ahrendts et al., 2011) in ADHD adults and in parieto-occipital areas in ADHD children (Filipek et al., 1997). In 22q11.2 deletion syndrome, a neurodevelopmental disorder caused by interstitial deletions of chromosome 22q11.2 and commonly associated with learning difficulties, specific cognitive deficits and high risk of neuropsychiatric disorders, the medial parietal occipital cortex is decreased in GMV (Campbell et al., 2006). Thus, more studies are needed to resolve these discrepancies and one should exercise caution comparing findings from neurotypical and clinical populations.

There are very few studies linking visual cortical functions to impulsivity. In an earlier study to quantify voxelwise response to visual stimulation, individuals with autism spectrum disorders (ASD) showed larger perifoveal population receptive field in most extrastriate cortex, as compared to neurotypical individuals (Schwarzkopf et al., 2014). Authors suggested that visual cortical functions in ASD may be characterized by extrastriate cortical hyperexcitability or differential attentional deployment. Another study examined the effects of d-amphetamine on visual neuronal responses in the superficial layers of the superior colliculus in cats (Grasse et al., 1993). The results showed that, within the first hour following intravenous d-amphetamine injection, the size of receptive field gradually increased and on average increased by 5.6-fold, likely as a result of suppressed surround inhibition. Thus, to pursue the clinical implications of the current findings, future studies are warranted to examine visual cortical responses and the effects of catecholaminergic agents on visual cortical responses in impulsive individuals and patients with ADHD.

## Limitations and Conclusions

Although suggesting a cerebral structural correlate in the parietal occipital areas, the current findings do not rule out a frontal top-down mechanism for impulse control (e.g., Hu and Li, 2012; Hu et al., 2016). Many studies in humans and non-human primates have shown top-down control of visual cortical activities and its interaction with stimulus-driven processes during spatial attention and target identification (Desimone and Duncan, 1995; Kastner and Ungerleider, 2000; Corbetta and Shulman, 2002; Noudoost et al., 2010; Macaluso and Doricchi, 2013; Vossel et al., 2014). For instance, in a visuospatial task where an auditory cue primes attention to the target location, activities of the frontal eye field and intraparietal sulcus Granger causes visual cortical activities, in association with improved performance (Bressler et al., 2008). Further, we did not map the visual cortical activities or examine attention in the MR cohort; thus, the functional implications of altered visual cortical GMV that represents the lower peripheral visual field need to be investigated in future work.

A recent study reported higher white matter integrity in association with simulated risky driving but not personality traits, suggesting the importance of complementing questionnaire assessment with behavioral tasks that mimic real-life decision making (Kwon et al., 2014). Outcome measures from many laboratory tasks including the stop signal paradigms may be useful alternatives (Hendrick et al., 2010; Ide and Li, 2011; Bednarski et al., 2012). An additional consideration is the behavioral instrument used to evaluate impulsive personality. While the BIS-11 is widely used to study impulsivity in health and illness, a number of other instruments capture different dimensions of impulsive personality, such as the urgency, (lack of) premeditation, (lack of) perseverance and sensation seeking or UPPS scale (Whiteside and Lynam, 2003) and emotion-based rash impulsivity (Muhlert and Lawrence, 2015). Indeed, studies have reported divergent results with respect to these instruments. For instance, in chronic cocaine users, Barratt impulsivity is associated with decreased GMV of bilateral ventromedial prefrontal cortex (Qiu et al., 2014), but UPPS is correlated positively with GMV in the left inferior/middle frontal gyrus (Moreno-López et al., 2012a). Finally, the subjects who participated in the MR and behavioral experiments differed in demographic characteristics. Thus, the extent to which behavioral and imaging findings can be associated is limited by this consideration. We only recruited 10 subjects in the behavioral experiment, so these findings should be considered as tentative. It is also important to emphasize that participants were screened out of clinical conditions, so the findings are to be considered specific to individuals within a moderate range of BIS scores.

In summary, we reported increased GMV in parietal occipital areas in healthy young adults with higher Barratt impulsivity. This new finding suggests the importance of attentional dysfunction as an underlying process of this impulsive personality trait (Li et al., 2005) and may have particular relevance to understanding the etiology of ADHD (Amso and Scerif, 2015) and impulse control disorder.

## Author Contributions

C-SRL conceived and designed the experiments; JSI, HCT, C-TY and Y-CT performed the experiments; JSI, HCT and Y-CT analyzed the data; JSI, HCT, C-TY, Y-CT and C-SRL contributed to the writing of the manuscript.

## Conflict of Interest Statement

The authors declare that the research was conducted in the absence of any commercial or financial relationships that could be construed as a potential conflict of interest.

## Footnotes

^1^http://dbm.neuro.uni-jena.de/vbm/

^2^http://www.brain-development.org/
